# Top-class women’s soccer performance: peak demands and distribution of the match activities relative to maximal intensities during official matches

**DOI:** 10.5114/biolsport.2024.129477

**Published:** 2023-08-08

**Authors:** Andreas Riboli, Lorenzo Francini, Emanuele Rossi, Andrea Caronti, Lorenzo Boldrini, Stefano Mazzoni

**Affiliations:** 1 MilanLab Research Department, AC Milan S.p.A., Milan, Italy; 2 Department of Biomedical Sciences for Health, Università degli Studi di Milano, Milan, Italy; 3 Isokinetic Medical Group, FIFA Medical Centre of Excellence, Milan, Italy

**Keywords:** Team sports, Football, Training load, Coaching, High intensity training

## Abstract

The aims of the current study were to determine the most demanding passages of match play (MDP) and the distribution of match activities relative to maximum intensities during official matches in top-class women soccer players. Twenty-eight women players competing in European championship and international UEFA competitions were monitored during 38 official matches (277 individual samples). Maximum relative (m · min^−1^) total distance (TD), high-speed running (HSRD), very high-speed running (VHSRD), sprint, acceleration and deceleration distances were calculated across different durations (1–5, 10, 15, 90 min) using a rolling average analysis. Maximum intensities (1-min_peak_) were used as the reference value to determine the distribution of relative intensity across the whole-match demands (90-min_avg_). Time and distance higher than 90-min_avg_ (> 90-min_avg_) were also calculated. MDP showed *moderate* to *very large* [effect size (ES): 0.63/5.20] differences between 1-min_peak_ vs all durations for each parameter. The relative (m · min^−1^) 1-min_peak_ was greater than 90-min_avg_ of about +63% for TD, +358% for HSRD, +969% for VHSRD, +2785% for sprint, +1216% for acceleration, and +768% for deceleration. The total distance covered > 90-min_avg_ was ~66.6% of the total distance covered during the 90-min_avg_ for TD, ~84.8% for HSRD, ~97.4% for VHSRD, ~100% for sprint, ~99.1% for acceleration and ~98.2% for deceleration. The relative distance > 90-min_avg_ was higher (*P* < 0.05) than the 90-min_avg_ for each metric (ES: 2.22 to 7.58; *very large*). The present results may help coaches and sport scientists to replicate the peak demands during training routine in top-class women soccer players.

## INTRODUCTION

Nowadays, the popularity of women’s soccer is growing, and the understanding of the official match-play demand is crucial for high-performance development through an evidence-based decision-making process [1]. Different tracking technologies (e.g. global positioning system, semi-automatic video-analysis, etc.) are currently utilized to quantify the total distance (TD), the distance covered at different running speeds [2] and the distance covered while accelerating/decelerating during both training and matches [3]. In practice, the locomotor activities recorded during the matches are used to plan the training workload and as a reference for soccer-specific drills (e.g., small-sided games), technical-tactical drills and/or individual positional exercises [4–6].

The relative whole-match running distance (90-min_avg_) is usually utilized as a reference for player performance profiling and training prescriptions [6, 7]. Although match demands are affected by several factors such as playing position, the level of competition, the match-to-match variability and several others [8], it could be argued that top-class women players might cover a TD of ~9500 m to ~11200 m and a distance of ~800 m to ~1600 m between 15 and 20 km · h^−1^, ~500 m to ~900 m between 20 and 25 km · h^−1^, and ~190 m to ~250 m > 25 km · h^−1^ [9]. However, it has been previously reported that the 90-min_avg_ demands fail to fully account for the most demanding passages (MDP) of official match play [6 10, 11] determined across different time windows (e.g., 1, 2, 3, 5, 10 min periods) [11]. It may be responsible for underpreparing players for the MDP [11–13] during official matches. As such, the MDP analysis may help practitioners to contextualize the average [6] and peak [14] official match demands during the training routine [15]. The MDP during official matches were widely investigated in Serie A [11], French Ligue 1 [4], English Championship [16], reserve squad Spanish La Liga [3, 17] and Spanish La Liga 123 [18, 19] men soccer players. These highlighted that both 90-min_avg_ and the MDP across different durations should be considered as a reference for training prescriptions [20, 21]. The MDP across different #102/14) approved the durations may provide information to compare ball drills (e.g., smallsided games with or without goalkeepers, individual positional drills, etc.) of a different duration with the official match peak demands across a similar time window. As an example, it was recently shown how to replicate the 4-minute official match peak demands using ball drills in Italian Serie A men soccer players [14]. Unfortunately, in top-class women’s soccer, information about MDP has been determined solely in the 5-min period [9], but research findings about MDP of a different duration are still lacking. These latter are challenging issues for the comparison of the official match peak demands and the ball drills’ locomotor demands during training routine in women’s top-class soccer. This information may be crucial for both performance development and injury prevention purposes in top-class women’s soccer [14, 21].

Notwithstanding, the MDP theoretically occur only once or a few times during the game [10]. Therefore, conditioning for the MDP and whole match (90-min_avg_) relative intensity should be only a part of the overall periodized training programmes [14, 21]. Although 90-min_avg_, 1-min_peak_ and MDP across different time windows (e.g., 1, 2, 3, 5, 10 min periods) could guide the training prescription [6, 11, 14], also the distribution of match activities relative to the maximum intensities has been recently reported [20] as a tool to reduce the gap between training and official match demands. The distribution analysis investigates the demands of each minute from the most demanding minute (i.e. 1-min_peak_) to the less demanding minute during official matches and it may be used as a tool to have a more comprehensive analysis of the individual official match demands [21], as recently demonstrated in Australian soccer [22], rugby league [22], Italian Serie A [21] and Spanish La Liga men soccer players [20]. To the best of our knowledge, also the distribution of match activities regarding peak demands has not been previously investigated in top-class women’s soccer.

Therefore, the current study aims to determine for the first time the 1-min_peak_, the MDP across different time windows and the distribution of match activities relative to the maximum intensities in top-class women soccer players. Additionally, it aims to determine the match-to-match variability in 1-min_peak_ *vs* 90-min_avg_ during official matches [23]. Lastly, the time spent and the distance covered at different percentages relative to the maximal match-play demands were calculated.

## MATERIALS AND METHODS

### Participants

Twenty-eight (*n = 28*) top-class women soccer players competing in European championship and international UEFA competitions were monitored during official matches across the 2019–2020 and 2020–2021 seasons. Goalkeepers were not included in the analysis. A total of 277 individual observations were collected. The number of individual matches varied among players (*n* = 9.9 ± 5.3, range: 2–18). The Ethics Committee of the University of Milan (protocol #102/14) approved the study. It was performed in accordance with the principles of the Declaration of Helsinki (1975).

### Experimental design

Data were collected during 38 official home matches. A 18 Hz Global Positioning System unit (GPEXE Pro2, Exelio SRL, Italy, firmware version 0.13) was used to collect data during official matches [24]. Each device was turned on at least 15 min before each session to allow for acquisition of the satellite signal [6]. To reduce the inter-unit differences, each player wore the same unit for every match over the whole investigation [6]. The system has previously been shown to provide valid and reliable measurements of the match activity in soccer [24, 25].

### Procedures

Following the completion of each match (~90 min), each file was trimmed so that only data recorded when the player was on the field for at least 85 min were included for further analysis [11]. Data were exported into a customized Microsoft Excel spreadsheet (Microsoft, Redmond, USA). A customized spreadsheet was used to allow analysis of relative distance covered (m · min^−1^) in the following categories: total distance (TD), high-speed running distance (HSRD, 15.1 to 20 km · h^−1^), very high-speed running distance (VHSRD, 20.1 to 24 km · h^−1^), sprint distance (sprint, > 24.1 km · h^−1^), distance with variations in running speed > 3 m · s^2^ (acceleration) and distance with variations in running speed < 3 m · s^2^ (deceleration) [6]. To assist in the development of velocity-based movement indicators, the rolling moving average was utilized to calculate the most demanding one-minute period (1-min_peak_) and the maximal locomotor demands across six other durations (2, 3, 4, 5, 10 and 15 min) for each player across each match [11, 12, 17]. To compare with the traditional metrics analysis, the distance over the whole match demand (90-min_avg_) was recorded and inserted into the data analysis. As previously proposed [20, 21], the 1-min_peak_ was then used as the reference value to determine the distribution of relative intensity across the whole match for all other rolling 1-minute periods. The match-to-match variability in 1-min_peak_ and 90-min_avg_ were calculated for TD, HSRD, VHSRD, sprint, acceleration and deceleration [11]. The time and distance higher than 90-min_avg_ (> 90-min_avg_) were calculated as the minutes or distance covered at intensity higher than the percentage of 1-min_peak_ corresponding to the average 90-min_avg_ [21].

### Statistical analysis

SPSS (version 26, IBM, USA) was used to perform the statistical analysis. A linear mixed model analysis was used to compare the effects of the duration of each period of match demands and the distribution of the match activities on the dependent parameter [11, 26]. The model used for each dependent parameter was with the duration of each period or with the distribution of match activities as independent fixed factors and random intercepts on the individual players. A log-likelihood ratio test was used to assess the goodness of fit of the models. Bonferroni’s correction was used for multiple comparison analysis. Between-matches coefficient of variation (CV) values were calculated for 1-min_peak_ and the 90-min_avg_ demands for TD, HSRD, VHSRD, sprint, acceleration and deceleration. Cohen’s *d* effect size (ES) with 95% confidence interval (CI) was used to describe the magnitude of the pairwise differences and interpreted as follows: < 0.20: *trivial*; 0.20–0.59: *small*; 0.60–1.19: *moderate*; 1.20–1.99: *large*; ≥ 2.00: *very large* [27]. Statistical significance was set at α < 0.05. Unless otherwise stated, all values are presented as mean (SD) as reported using descriptive statistics.

## RESULTS

*The most demanding passages of play across different durations* Table 1 shows the maximal locomotor demands for each duration (1 to 5, 10, 15, 90 min). For each variable, as the time-dependent period decreases, an increase in maximal relative locomotor demand was found (*P* < 0.05). Descriptive results with differences across all durations for relative TD, HSRD, VHSRD, sprint, acceleration and deceleration are presented in Table 1.

**TABLE 1:** The most demanding passage of match play for each metric during official matches for different time duration (1, 2, 3, 4, 5, 10, 15, 90-min). All data are reported as average (SD). 95% confidence intervals of the effect size were shown for the differences between 1-min *vs* all other time durations (horizontal direction).

	1-min	2-min	3-min	4-min	5-min	10-min	15-min	90-min	ES (95% CI)
TD	169.6 (15.4)	149.1 (12.8)	140.3 (11.4)	135.7 (10.7)	132.2 (10.6)	123.8 (10.3)	119.6 (9.8)	104.2 (8.5)	1.66 to 5.20
HSRD	56.2 (13.2)	39.0 (9.7)	32.0 (7.5)	28.4 (6.6)	26.3 (6.5)	20.7 (5.2)	18.5 (4.8)	12.3 (3.3)	1.49 to 4.51
VHSRD	31.2 (9.9)	18.9 (6.5)	14.2 (5.0)	12.0 (4.3)	10.5 (3.9)	7.2 (2.9)	6.0 (2.4)	2.9 (1.4)	1.56 to 3.96
Sprint	19.7 (13.2)	10.9 (7.7)	7.6 (5.6)	5.9 (4.4)	5.0 (3.9)	2.9 (2.4)	2.2 (1.9)	0.7 (0.4)	1.08 to 2.01
ACC	1.8 (0.6)	1.1 (0.4)	0.8 (0.3)	0.7 (0.3)	0.6 (0.3)	0.4 (0.2)	0.3 (0.2)	0.1 (0.0)	0.63 to 3.29
DEC	3.8 (1.3)	2.5 (0.9)	1.9 (0.7)	1.6 (0.6)	1.4 (0.6)	1.0 (0.4)	0.9 (0.4)	0.4 (0.2)	1.56 to 3.61

Abbreviations: TD, maximum relative total distance; HSRD, high-speed running distance; VHSRD, very high-speed running distance; Sprint, sprint distance; ACC, acceleration distance with velocity changes calculated using > 3 m · s^−2^; DEC, deceleration distance with velocity changes calculated using < 3 m · s^−2^.

**FIG. 1:**
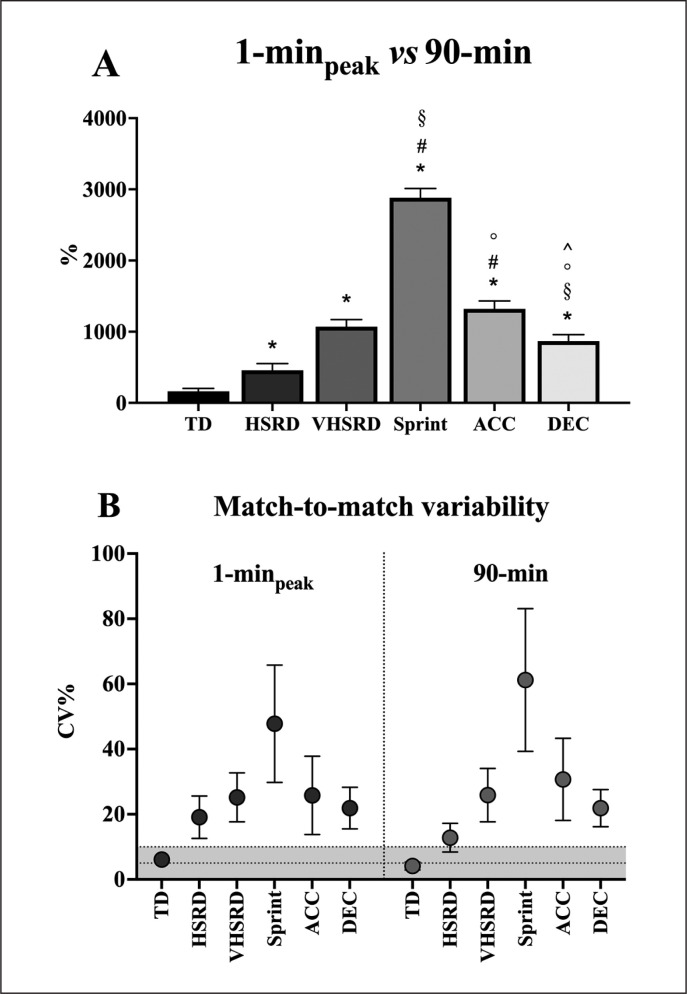
The 1-min_peak_ as percentage of the whole-match demands (90-min) (Panel A) and the match-to-match variability for both 1-min_peak_ and 90 min (Panel B) are shown for total distance (TD), high-speed running distance (HSRD), very high-speed running distance (VHSRD), sprint distance (SPR), acceleration (ACC) and deceleration (DEC). *P < 0.05 vs TD; ^#^*P* < 0.05 vs HSRD; ^§^*P* < 0.05 vs VHSRD; ^°^*P* < 0.05 vs sprint; ^^^*P* < 0.05 vs acceleration.

As shown in Figure 1 (Panel A), the magnitudes of the percentage differences between 1-min_peak_ and 90-min were sprint > acceleration = VHSRD > deceleration > HSRD > TD (ES: 2.11 to 28.2). As shown in Figure 1 (Panel B), the 1-min_peak_ performance showed ~6% to ~48% match-to-match variability for TD and sprint, respectively. Sprint variability was higher for 90-min than 1-min_peak_ (~61% *vs* 48%, respectively). No further difference in match-to-match variability between 1-min_peak_ and 90-min was found (Figure 1, Panel B).

**FIG. 2:**
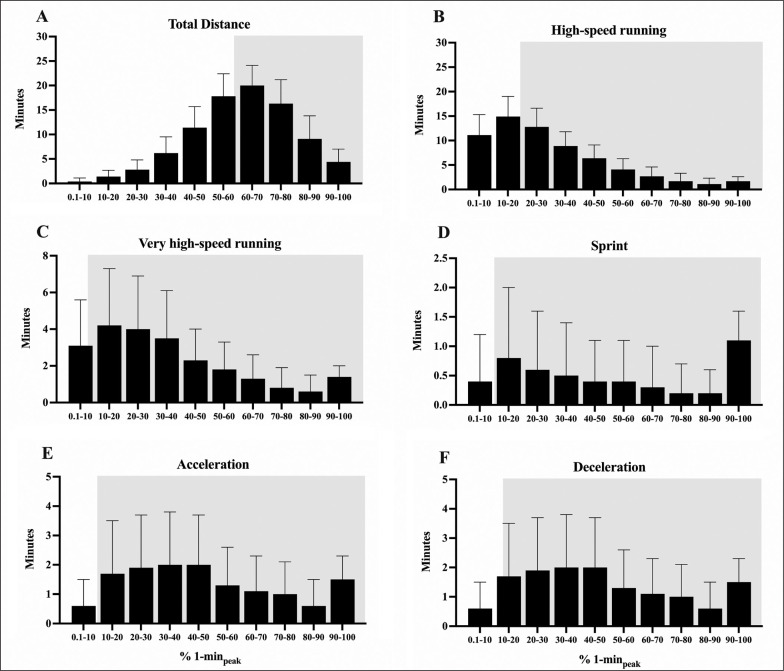
The time spent at different percentages (from 0–10% to 90–100%) of the peak demands recorded in 1 min (% 1-min_peak_) is shown for each metric. The grey area highlights the time spent at match activities higher than the average whole-match demands. Total distance: Panel **A**; high-speed running: Panel **B**; very-high speed running: Panel **C**; sprint: Panel **D**; acceleration: Panel **E**; deceleration: Panel **F**.

The distribution of the time spent at different percentages of 1-min_peak_ for TD, HSRD, VHSRD, sprint, acceleration and deceleration are presented in Figure 2. Main effects for distribution of match activities were found for each dependent parameter (*P* < 0.001).

**FIG. 3:**
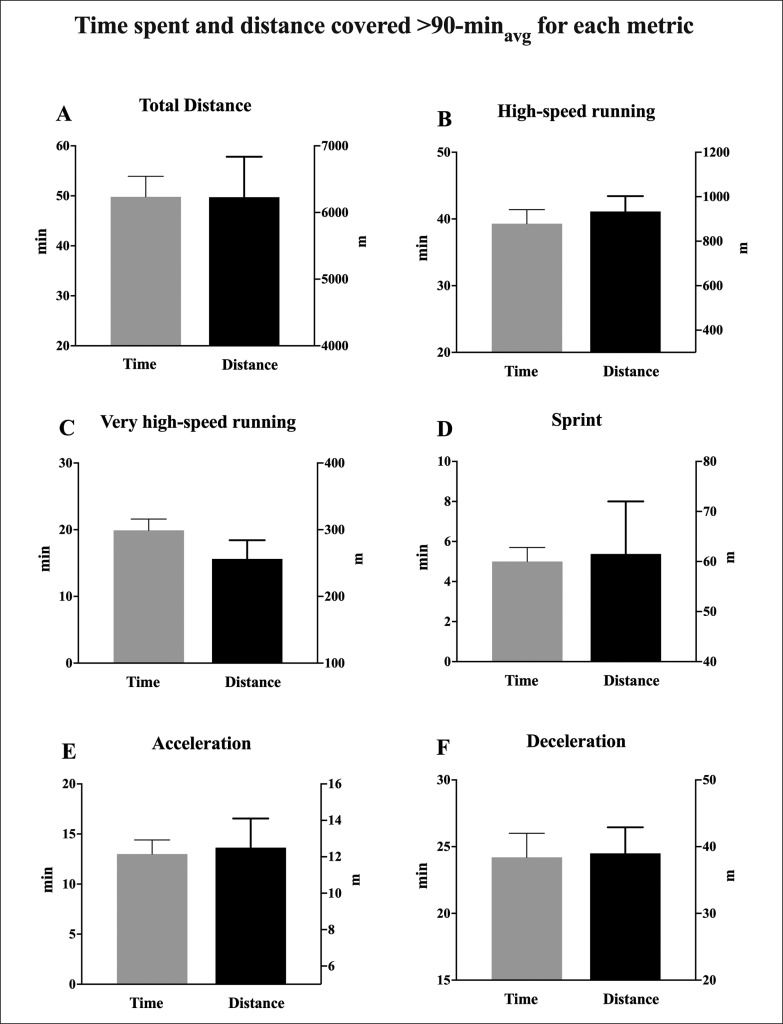
The time spent (min) and distance covered (m) at match activities higher than the average whole-match demand (90-min_avg_) are shown for each metric. Total distance: Panel **A**; high-speed running: Panel **B**; very-high speed running: Panel **C**; sprint: Panel **D**; acceleration: Panel **E**; deceleration: Panel **F**.

Figure 3 summarizes the time spent and the total distance covered > 90-min_avg_ for TD, HSRD, VHSRD, sprint, acceleration and deceleration; the percentage of the total distance covered > 90-min_avg_ than 90-min_avg_ was ~66.6(4.0)% for TD, ~84.8(1.9)% for HSRD, ~97.4(0.2)% for VHSRD, ~100(0.0)% for sprint, ~99.1(0.3)% for acceleration and ~98.2(0.5)% for deceleration; the relative distance covered at > 90-min_avg_ was higher (*P* < 0.05) than the relative 90-min_avg_ (ES: 2.22 to 7.58; *very large*) for each metric [TD: 125(9.1) m · min^−1^ vs 104.2(8.5) m · min^−1^; HSRD: 23.7(4.3) m · min^−1^ vs 12.3(3.3) m · min^−1^; VHSRD: 12.9(2.3) m · min^−1^ vs 2.9(1.4) m · min^−1^; sprint: 12.3(2.1) m · min^−1^ vs 0.7(0.4) m · min^−1^; acceleration: 1.0(0.4) m · min^−1^ vs 0.1(0.4) m · min^−1^; deceleration: 1.6(0.3) m · min^−1^ vs 0.4(0.2) m · min^−1^].

## DISCUSSION

The current study aimed to describe the 1-min_peak_, the MDP across different time windows, the distribution of match activities relative to the maximum intensities and the match-to-match variability in 1-min_peak_ and 90-min_avg_ during official matches in top-class women soccer players. For the first time, the 1-min_peak_, the MDP across different time windows and the distribution of match activities with regards to 1-min_peak_ determined during an official match have been described. Firstly, the locomotor activities calculated at 90-min_avg_ were much lower than 1-min_peak_ for each metric, especially for the high-intensity activities; interestingly, no differences in the match-tomatch variability between 90-min_avg_ and 1-min_peak_ were found except for sprint, with a lower variability in 1-min_peak_ than 90-min_avg_ (~61% vs 48%, for 90-min_avg_ and 1-min_peak_, respectively). Secondly, most of the locomotor activities occur at an intensity > 90-min_avg_. Thirdly, except for the *small* difference for acceleration, the relative distance > 90-min_avg_ was significantly higher (ES: *large* to *very large*) than the 90-min_avg_ for each metric.

For the first time, the present findings show the official match peak demands during top-class women’s soccer. As previously described in men’s soccer, also the current study in women’s soccer highlights that the locomotor demands for TD, HSRD, VHSRD, acceleration and deceleration were higher during shorter time windows. As such, the 1-min_peak_ locomotor demands are higher than the other longer time periods (e.g. 1-min_peak_ vs 4-, 5-, 10-, 15-min periods), and especially than 90-min_avg_. Similar results were previously described in Italian Serie A [11], French Ligue 1 [4], Spanish La Liga [20], Spanish La Liga 123 [18, 28] and Youth/Adult Premier league [29] male soccer players. Comparisons with previous findings are challenging due to the lack of previous studies about peak demands across different durations in top-class women’s soccer. This is the first study that identify the 1-, 2-, 3-, 4-, 5-, 10-, 15-min peak demands for different metrics in top-class women’s soccer. Comparing match performance of Italian Serie A women vs previous results in Italian Serie A men [11] soccer players, the current results highlights 1-min_peak_ demands of ~169 vs ~188 m · min^−1^ for TD, ~56 *vs* ~58 m · min^−1^ for HSRD, ~31 vs ~37 m · min^−1^ for VHSRD, ~19 vs ~42 m · min^−1^ for sprint and ~6 vs ~32 m · min^−1^ for acceleration+deceleration in women vs men, respectively. Therefore, women players covered lower TD, similar HSRD, lower VHSRD and a significantly lower sprint and acceleration+deceleration distance than men soccer players of a similar Italian Serie A population. Similarly, the present findings show a lower total distance (i.e. ~169 vs ~190 m · min^−1^) and a lower high-speed running (i.e. ~51 vs ~59 m · min^−1^) in Italian Serie A women player than English Championship men players [29]. The differences with regards to men soccer are probably due to a lower neuromuscular ability (i.e. muscular strength, power, etc.) in women, as previously reported [30]. Lower cardiorespiratory [31] and neuromuscular [30] abilities were previously demonstrated in women than men across different sports [30, 31] including elite soccer [30, 31]. Therefore, the lower distance covered at the highest speed and/or acceleration/deceleration thresholds in women than men could be explained by between-gender neuromuscular [30], cardiorespiratory [30, 32] and anthropometric [32] differences. Unfortunately, the lack information about peak locomotor demands in top-class women’s soccer challenges the comparisons of peak demands across a similar women population.

For the first time here, the current findings describe the distribution of match activities with regards to 1-min_peak_. The average (90-min) locomotor match demand was about ~60% and ~20% of the 1-min_peak_ for TD and HSR, respectively, while it was ~10% for VHSR, sprint, acceleration and deceleration. As such, when a woman soccer player plays a match, most running activities are covered at intensity higher than 90-min_avg_ official-match demands, especially for high-speed running, sprint and acceleration/deceleration activities. Since the current findings in women’s soccer are shown for the first time here, comparisons with previous results in women’s soccer are challenging. Comparing the current information with previous findings in male Italian Serie A [21] and male Spanish La Liga 123 [20] soccer players, a similar distribution of match demands for different metrics was found. However, the gap between 1-min_peak_ and 90-min_avg_ is larger in women than men for HSR, VHSR, acceleration and deceleration. For HSR, the 90-min_avg_ was at ~20% or ~30% than 1-min_peak_ in women or men [21], respectively; for VHSR, acceleration and deceleration the 90-min_avg_ was at ~10% or ~20% than 1-min_peak_ in women or men [33], respectively. Therefore, coaches and sport scientists should consider the current results for preparing women players for peak locomotor demands determined during the official matches. Interestingly, the official match demands require an intensity higher than 90-min_avg_ with ~6226 m covered in ~50 min for TD, ~933 m covered in ~39 min for HSR, ~256 m covered in ~20 min for VHSR, ~61 m covered in ~5 min for sprint, ~12.5 m covered in ~13 min for acceleration and ~39 m covered in ~24 min for deceleration. These findings suggest that the official match locomotor demands are often higher than the 90-min_avg_. Therefore, the 90-min_avg_ official match demands should not be considered alone as a reference for training prescriptions because it may underestimate the locomotor demands during official matches. Similar findings have been reported in Italian Serie A [21] and Spanish La Liga [20] male soccer players; similarly, it was suggested that the average match demands did not effectively reflect the locomotor match demands. Other comparisons with previous research findings are challenging because no previous studies investigated the distribution of match activities during official matches in top-class women’s players. Coaches and sport scientists should consider the intensity higher > 90-min_avg_ as a possible reference for the whole training intensity across training periodization. In detail, the 90-min_avg_ vs the > 90-min_avg_ official match demands was ~104 vs ~124 m · min^−1^ for TD, ~12 vs ~24 m · min^−1^ for HSR, ~2.9 vs ~12.8 m · min^−1^ for VHSR, ~0.7 vs ~12.3 m · min^−1^ for sprint, ~0.1 vs ~1.0 m · min^−1^ for acceleration and ~0.4 vs ~1.6 m · min^−1^ for deceleration. Therefore, practitioners should consider intensifying the training demands to cope with the official match demands > 90-min_avg_ for preparing women players for match performance demands. Moreover, as reported above, during most conditioning training sessions, the stake-holders could try to replicate the > 90-min_avg_ (i.e., as a reference for the full session demands) and the peak demands across different durations (i.e., as a reference for sport-specific drills and/or running-based exercises) to prepare the women players for the average and peak demands of the competition. These findings further concern the > 90-min_avg_ match-play demands and the peak demands for both performance development [14] and injury prevention [34] purposes.

The current findings come with some limitations: i) this is a team study, so between-squad differences (e.g., formation, style of play, style of coaching and cardiorespiratory or neuromuscular individual player characteristics, etc.) could affect the current results; ii) the locomotor metric utilized in this study was arbitrary and not individualized, affecting the possibility to highlight also the locomotor demands with regards to the maximal individual capacities. As such, it should be acknowledged that the maximal individual capacity in different metrics (e.g., VHSR, sprint, acceleration, deceleration, etc.) can exceed the maximal positional match-play requirements. For example, locomotor load > 90-min_avg_ or 1-min_peak_ for a given player could be lower than her VHSR, sprint, acceleration or deceleration maximal capacity. iv) Despite the current results open to the opportunity to contextualize the maximal match-play locomotor demands in women’s soccer, the locomotor load > 90-min_avg_ or 1-min_peak_ did not take into account the cardiorespiratory and metabolic individual capacity [23] and it could lead to lower the training stimuli; coupling locomotor and physiological demands during training routine is suggested for appropriate player’s conditioning [6, 11]. Therefore, soccer-specific exercises (e.g., small- or large-side games) [6], position-specific drills [35] and/or individualized running based exercises [36] with the aims to recreate or overload locomotor load from > 90-min_avg_ to 1-min_peak_ should be coupled with soccer-specific, positional-specific or individual running-based exercises near to the maximal individual aerobic [37], anaerobic [38] and neuromuscular [39] capacity for maximizing the performance development in top-class women soccer players. Therefore, despite the current limitations, these findings open several new future perspectives in top-class women’s soccer.

The present findings have several practical applications. In practice, coaches and sport scientists could utilize the MDP determined during the official match as a reference for training prescriptions and performance development during daily on-field routine. Although the 90-min_avg_ demands are usually considered as a reference for training prescription [6], the current findings further suggest that the 90-min_avg_ could not be the only reference for prescribing the intensity for the full training demands; an intensity > 90-min_avg_ should be considered for the full training session demands, as previously reported in male’s soccer [14, 20]. As an example, at match-day minus 4 or minus 3, coaches and sport scientists could consider replicating the MDP, the 90-min_avg_ and the > 90-min_avg_ of official matches using small-sided games in specific pitch sizes [14] for mimicking and/or overloading the individual official match peak locomotor demands. Additionally, when practitioners aim to prescribe ball drills of different durations, the 90-min_avg_ and/or the > 90-min_avg_ demands may underestimate the MDP of the official matches, possibly unpreparing the women players for the peak demands of the competition [4, 10, 11]. Therefore, coaches and sport scientists could manage the intensity of ball-drills of different durations using the 1-, 2-, 3-, 4-, 5-, 10-, 15-min peak demands as a reference to replicate the MDP of a similar duration determined during the official matches. For example, the manipulation of the relative pitch sizes during small-sided games may help to replicate the official match 90-min_avg_ [6] and/or the peak demands (e.g. 4-minPeak) [14] for each metric, especially for HSRD and sprint. Using small-sided games, it has been recently shown that a relative area per player of ~350 m^2^ · player should be utilized for replicating the 4-minpeak determined during official matches in elite Serie A men soccer players [14]. Conversely, when small-sided games have to be played on small pitches for overloading the technical demands [40], supplemental individual exercises (i.e. running base and/or positional exercises) should be considered to prepare the players for the individual official match peak locomotor demands, especially for high-speed to sprint running. This approach may help to maximize the performance development in top-class women’s soccer. Since VHSRD and sprint have been previously proposed as effective tools for injury prevention purposes [34], preparing players for the maximal match-play demands, especially for VHSRD and sprint, may help to optimize the individual exposure to the worst-case scenario during the official matches. This may help to reduce the gap between training and official match demands, helping to maximize the performance development and possibly positively affecting injury prevention in women’s soccer [34, 41].

## CONCLUSIONS

The official match peak demand and the distribution of match activities with regards to 1-min_peak_ have been described. Firstly, the locomotor activities calculated at 90-min_avg_ were much lower than 1-min_peak_, especially for the high-intensity activities; interestingly, no differences in the match-to-match variability between 90-min and 1-min_peak_ were found. Secondly, most of the locomotor activities occur at an intensity > 90-min_avg_. Thirdly, the relative distance > 90-min_avg_ was *very largely* than the 90-min_avg_ for each metric. Therefore, the current results should be considered as a reference for training prescription with the aim to prepare top-class women soccer players for the maximal locomotor demands during official matches.
